# Potential evidence for biotype-specific chemokine profile following BVDV infection of bovine macrophages

**DOI:** 10.1016/j.vetimm.2012.08.009

**Published:** 2012-11-15

**Authors:** Stephen Burr, Carole Thomas, Joe Brownlie, Victoria Offord, Tracey J. Coffey, Dirk Werling

**Affiliations:** a Royal Veterinary College, Department of Pathology and Infectious Diseases, Hawkshead Lane, Hatfield AL9 7TA, UK; b School of Veterinary Medicine and Science, Faculty of Medicine & Health Sciences, University of Nottingham, Sutton Bonington LE12 5RD, UK

**Keywords:** Chemokines, BVDV, Microarray

## Abstract

Chemokines play a key role in initiating the innate and subsequently adaptive immune response by recruiting immune cells to the site of an infection. Monocytes/macrophages (MØ) are part of the first line of defence against invading pathogens, and have been shown to release a variety of chemokines in response to infection. Here, we reveal the early transcriptional response of MØ to infection with cytopathogenic (cp) and non-cytopathogenic (ncp) bovine viral diarrhoea strains (BVDV). We demonstrate up-regulation of several key chemokines of the CCL and CXCL families in MØ exposed to cpBVDV, but not ncpBVDV. In contrast, infection of MØ with ncpBVDV led to down-regulation of chemokine mRNA expression compared to uninfected cells. Data suggest that ncpBVDV can shut down production of several key chemokines that play crucial roles in the immune response to infection. This study helps to further our understanding of the pathogenesis of BVDV infection, highlighting biotype-specific cellular responses.

## 1 Introduction

The pestiviruses, bovine viral diarrhoea virus (BVDV), classical swine fever virus and border disease virus of sheep, together with the flaviviruses and hepatitis C virus, are a closely related group of small enveloped viruses, the *Flaviviridae*, with a single-stranded, positive-sense RNA genome of approximately 12.5 kb. The RNA is translated into a single virus polyprotein that is processed by both viral and host proteases to either 11 or 12 virus polypeptides, dependent on the virus biotype. The viruses are generally non-cytopathogenic (ncp), although BVDV associated with the development of mucosal disease in persistently infected (PI) animals is a cytopathogenic (cp) biotype, producing an additional virus polypeptide, NS3, which represents the C-terminal two thirds of NS2-3 present in ncpBVDV strains (Donis and Dubovi, 1987).

Several groups have recently attributed the persistent infection to the ability of ncpBVDV to interfere with the induction of type-I interferon (IFN) production in macrophages (MØ) (Baigent et al., 2004, 2002; Charleston et al., 2001; Schweizer and Peterhans, 2001). This lack of IFN induction and the failure to induce apoptosis might be advantages for the survival of the virus (Schweizer and Peterhans, 1999, 2001). We and others have previously investigated the effect of a BVDV infection on molecules of the innate immune systems (Franchini et al., 2006; Werling et al., 2005). In addition to the activation of the type-I IFN system early after viral entry, one of the first responses seen in infected cells is the production of chemokines (Lim and Murphy, 2011). The chemokine system has been shown to play a crucial role in both homeostasis and disease mechanisms. These key proteins have been highlighted in the analysis of several economically important bovine diseases, including tuberculosis (Widdison and Coffey, 2011; Widdison et al., 2010, 2009). The system is complex, and relies on the chemokine ligand binding to its receptor, with additional complexity arising from the fact that multiple chemokines can bind a single receptor and vice versa. Both the chemokines and their receptors are grouped into four families, CC, CXC, XC, CX_3_C chemokines, depending on the location of C terminal cysteine residues in the chemokines with the receptor classification based on the chemokine family they bind.

For some members of the *Flaviviridae*, such as Dengue virus and Hepatitis C virus, the importance of chemokines in viral persistence, pathogenesis and infection of immune cell subsets has been clearly demonstrated (Chen and Wang, 2002; Gandini et al., 2011; Heydtmann and Adams, 2009; Kang and Shin, 2011; Kelley et al., 2011; Larrubia et al., 2008). More recently, ncpBVDV infection has been shown to impact on CXCR4 and CXCL12 mRNA expression (Weiner et al., 2012). To investigate the potential for a biotype-specific bovine MØ response to infection, we compared the response to cp and ncpBVDV, with specific emphasis on the effects on chemokine production.

## 2 Materials and methods

### 2.1 Animals and cell culture

Blood was collected from Holstein Friesian cattle in accordance with Home Office regulations. For generation of monocyte-derived macrophages (MØ), PBMC isolated by an adapted Histopaque procedure were sealed in Teflon bags (10–20 ml, 4 × 10^6^ PBMC ml^−1^) as described previously (Werling et al., 2004), and cultured for 6–8 days at 37 °C in a humidified atmosphere of 5% CO_2_. The medium was RPMI 1640 containing 10 mM HEPES (pH 7.4), 100 IU ml^−1^ penicillin, 100 μg ml^−1^ streptomycin, 1% (v/v) nonessential amino acids for MEM (Invitrogen, UK), 0.4% (v/v) vitamin solution for MEM (Invitrogen), 2 μM glutamine (Invitrogen), 40 μg ml^−1^ folic acid, 1 mM sodium pyruvate (Invitrogen), 2.5 μM amphotericin B (Invitrogen) and 15% heat-inactivated FCS (Invitrogen). During culture, monocytes matured to non-activated MØ, which optimally responded to LPS by NO generation and TNF production (Jungi et al., 1996). From the PBMC, MØ were purified by selective adherence to microtiter plates wells for 3 h. After washing, viability was assessed by Trypan blue exclusion and was normally above 98%.

### 2.2 Cultivation of bovine viral diarrhoea virus

Both the cp and ncp PEC515 isolates of BVDV were grown in primary foetal bovine lung cells (FBLs) cultured in DMEM (Invitrogen) containing 1% antibiotic/antimycotic (Invitrogen) and 2% FCS (PAA, Austria). Serial dilutions of each virus were cultured on FBLs for five days. This was followed by virus titre (TCID_50_) determination using microscopic examination for cytopathic effect or using the immunoperoxidase assay on fixed cells (ncp) to detect the presence of intracellular virus. In brief, cells were acetone fixed and labelled with an anti-BVDV hyperimmune serum V182 (Brownlie, personal communication), washed and counter-labelled with an HRP-conjugated anti-bovine secondary antibody (Sigma). The presence of virus was then visualised with AEC colour substrate (Sigma) under a light microscope. In both cases, the virus titre was calculated using the formula from Spearman and Karber (Villegas, 1998).

### 2.3 Viral Infection of macrophages and RNA extraction

MØ were infected with either cp or ncp PEC515 at a MOI of 0.1 for 1 h. Thereafter, cells were washed once with cold PBS, before lysis of the cells. Total RNA was isolated from infected cells after 6 h of incubation using the total RNA extraction kit (Qiagen), followed by DNAseI treatment (Ambion). The RNA was analysed on a 1% agarose denaturing gel to assess quality and potential degradation.

### 2.4 cDNA generation and microarray analysis

Infections were set up as described above using blood-derived MØ from three separate animals. Cells from each animal were infected with either cpPEC515BVDV, ncpPEC5154 BVDV or a mock infection, providing three RNA samples from each animal. RNA was extracted from these cells using the Qiagen RNeasy Minikit. Initially the experimental design was to use two microarray slides per set of samples, comparing the RNA from the cp-infected cells to that from the ncp-infected and conducting a dye swap for each animal. These RNA samples were prepared for use by first creating amine-modified cDNA using the Superscript III Fluorescent Labelling Kit (Invitrogen). After purification and precipitation of the cDNA, it was linked to Cy3 and Cy5 dyes. The labelled samples were quantified on a spectrophotometer and the labelling efficiency of the dye was determined. Since the amount of RNA in the samples was low, an RNA amplification step was included. The Ambion Amino Allyl MessageAmp II aRNA Amplification Kit was used according to manufacturer's instructions. Briefly, first strand cDNA was produced from the RNA samples by reverse transcription; this was followed by second strand cDNA synthesis by DNA polymerase. The double stranded cDNA was then purified and used to transcribe biotin-labelled aRNA. Once purified the aRNA was labelled with Alexa Fluor dyes (555/647), and then purified using the Qiagen MinElute Clean Up Kit. Samples were run on ARK Genomics 17k Bovine cDNA array, with hybridisation performed using a TECAN HS400™ Pro hybridisation machine. Slides were scanned on an Axon GenePix 4000A scanner using GenePix Pro 6 software. Data were subsequently analysed using the Genepix Pro 6 software and followed by R (version2.5.0) and Limma (version 2.9.17) software. Normalisation of data was performed using the Lowess method (Cleveland, 1981; Cleveland and Devlin, 1988).

## 3 Results and discussion

The aim of this study was to identify potential biotype-specific responses of BVDV infection of MØ, comparing production of chemokines and identifying potential downstream effects on subsequent immune cell recruitment.

**Fig. 1:**
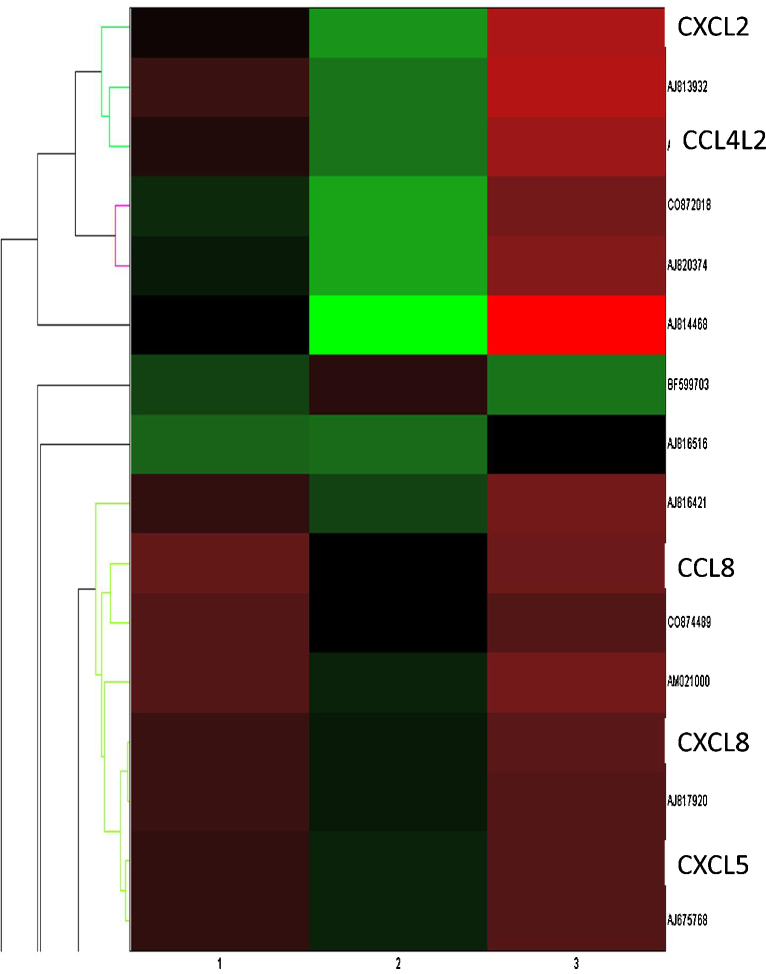
Clustergram graphically depicting hierarchical clustering analysis of expressed chemokine genes between cpBVDV, ncpBVDV and uninfected bovine macrophages. Each row represents a specific gene, each column represents a treatment. A representative analysis of three repeats is shown. Green represents genes up-regulated by treatment, red represents down-regulated genes, with the intensity of colour indicating the extend of up- or down-regulation. Chemokine-genes identified are shown, for other genes in this section of the array, gene-bank accession numbers are shown. (For interpretation of the references to color in this figure caption, the reader is referred to the web version of the article.)

**Fig. 2:**
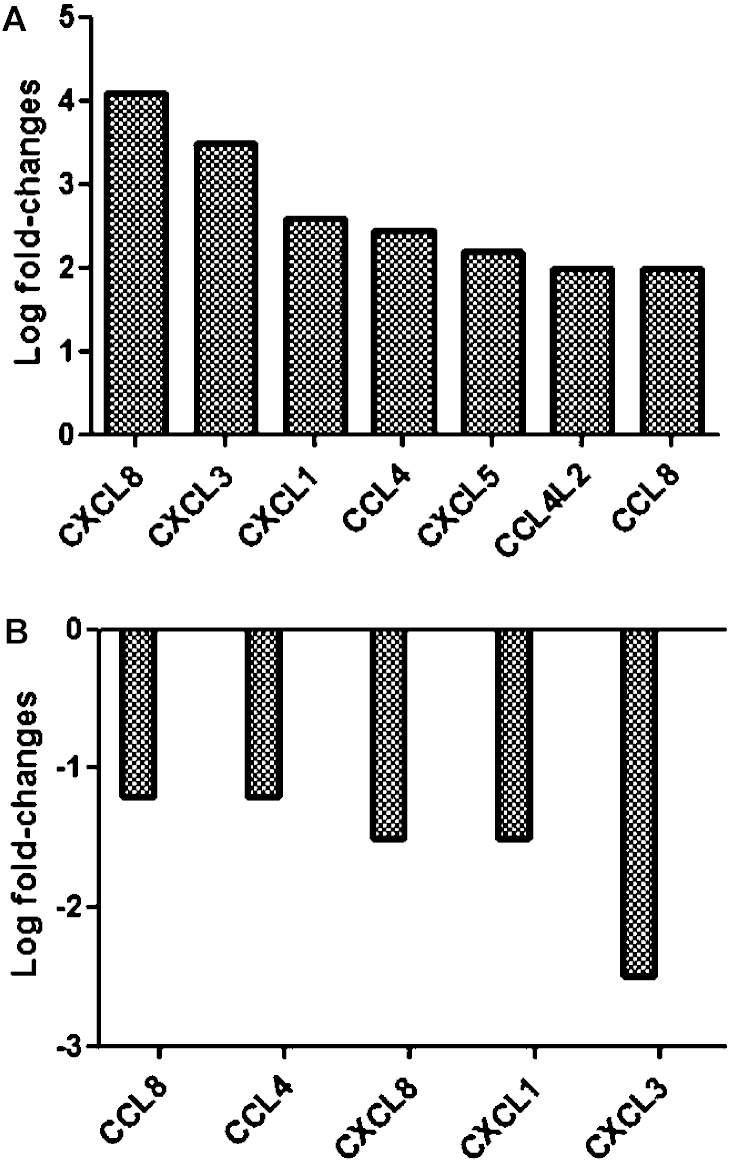
Fold expression differences for identified chemokines between mRNA isolated from cpBVDV and ncpBVDV infected macrophages (A), and ncpBVDV and uninfected macrophages (B) based on microarray analysis.

Fig. 1 shows the results of hierarchical clustering of differentially expressed chemokine genes, illustrating that infection of MØ with the cpBVDV strain induced a greater number of differentially expressed chemokine genes, compared to both to ncpBVDV-infected and uninfected MØ. Interestingly, only two genes with a significant log_2_ fold change were found in the cpBVDV versus uninfected data set (data not shown). The first of these genes, CCL8 (former name monocyte chemotactic protein 2 (MCP-2)), had an adjusted *P* value >0.05 and therefore cannot be considered statistically significant. The function of the second gene is currently unknown. In contrast, more genes were up-regulated when comparing cpBVDV versus ncpBVDV-infected cells (Fig. 2A). Infection of bovine MØ with cpBVDV resulted in the up-regulation of numerous chemokines, including CXCL3 (former name Macrophage Inflammatory Protein 2β), CCL4 (former name Macrophage Inflammatory Protein 1β) and CCL8. As all these chemokines have been described to stimulate migration and intra-vascular adhesion of monocytes, their upregulation may support two different scenarios. Firstly, assuming that this upregulation on the mRNA level results in an appropriate immune response, all chemokines induced by cpBVDV infection of MØ should enable the host to cope faster and more efficient with the invading pathogen. Secondly, further recruitment of (innate) immune cells to the side of infection may facilitate the spread of cpBVDV within the host.

As mentioned, all chemokines bind to chemokine receptors, a family of closely related 7-transmembrane G-protein coupled receptor molecules (Holmes et al., 1991). Within the present study, cpBVDV infection of MØ mainly enhanced expression of chemokines binding to CCR5 (CCL4, CCL4L2, CCL8) and CXCR2. CCR5 is considered as a co-receptor for the entry of monocyte-trophic HIV strains, and antagonists for this receptor are currently tested for their effects in preventing further infections of MØ in the human system. In addition, it is also expressed on endothelial cells as well as MØ. Thus, it is tempting to speculate that this receptor may have a similar function in cpBVDV infection of endothelial cells as in MØ, especially as all three chemokines are attracting further MØ. CCL4 is known to be produced by monocytes, activated T- and B-lymphocytes and by natural killer (NK) cells (Salazar-Mather and Hokeness, 2003, 2006). CCL4 and CCL4L2 have a strong chemotactic effect on lymphocytes and monocytes in particular; increasing trans-endothelial migration and activation of these cell types (Menten et al., 2002). CCL8 is expressed by a wide range of cells and is a chemoattractant for monocytes, lymphocytes, basophils, eosinophils and natural killer cells (Maghazachi, 2010). In contrast, CXCR2 is a receptor for all CXCL chemokines upregulated following cpBVDV infection of MØ. The main function of CXCL chemokines is to attract granulocytes to the foci of infection, subsequently stimulating reactive oxygen production by these cells. Binding of CXCL chemokines to this receptor induces chemotaxis, degranulation and upregulation of intracellular Ca^2+^ levels as well as being angiogenic (Busch-Petersen, 2006; Reutershan, 2006). Secretion of CXCL chemokines, such as CXCL1, CXCL2 and CXCL8 will therefore result in movement of neutrophils to the foci of infection and also increase neutrophil activity (Sadik et al., 2011).

In contrast, ncpBVDV infection resulted in down-regulation of these chemokines (Fig. 2B). The negative effects of ncpBVDV infection on the chemotactic activity of bovine MØ was documented over 30 years ago (Ketelsen et al., 1979); however, these observations were never followed up with the development of new technologies. As chemokines are produced very early on in the immune response, helping to target the immune response to the infected area as well as helping to initiate the acquired immune system, it is possible that ncpBVDV actively suppression chemokines production to dampen the early innate immune response and enhance its survival. This, in combination with the known effects on the type I IFN production (Gibson et al., 2011), would form a very effective evasion of the early immune response. Such inhibition in the initial stages could delay or prevent the establishment of an appropriate immune response, and may facilitate ncpBVDV infection with limited associated host response. However, to verify this hypothesis, more cp/ncp BVDV pairs need to be tested.
